# PNA5, A Novel Mas Receptor Agonist, Improves Neurovascular and Blood-Brain-Barrier Function in a Mouse Model of Vascular Cognitive Impairment and Dementia

**DOI:** 10.14336/AD.2023.0928

**Published:** 2024-08-01

**Authors:** Christina Hoyer-Kimura, Meredith Hay, John P Konhilas, Helena W Morrison, Methawasin Methajit, Joshua Strom, Robin Polt, Victoria Salcedo, Joshua P Fricks, Anjna Kalya, Paulo W Pires

**Affiliations:** ^1^Department of Physiology, The University of Arizona, Tucson, AZ 85724, USA.; ^2^Evelyn F. McKnight Brain Institute, The University of Arizona, Tucson, AZ 85724, USA.; ^3^College of Nursing, The University of Arizona, Tucson, AZ 85724, USA.; ^4^Department of Cellular and Molecular Medicine, The University of Arizona, Tucson, AZ 85724, USA.; ^5^Department of Chemistry and Biochemistry, The University of Arizona, Tucson, AZ 85724, USA.; ^6^ProNeurogen, Inc, Tucson, AZ, USA

**Keywords:** vascular dementia, PNA5, VCID, microglia, blood-brain-barrier, brain-blood-flow

## Abstract

It is well established that decreased brain blood flow, increased reactive oxygen species production (ROS), and pro-inflammatory mechanisms accelerate neurodegenerative disease progressions, including vascular cognitive impairment and dementia (VCID). Previous studies in our laboratory have shown that our novel glycosylated Angiotensin-(1-7) Mas receptor agonist PNA5 reverses cognitive deficits, decreases ROS production, and inhibits inflammatory cytokine production in our preclinical mouse model of VCID that is induced by chronic heart failure (VCID-HF). In the present study, the effects of VCID-HF and treatment with PNA5 on microglia activation, blood-brain-barrier (BBB) integrity, and neurovascular coupling were assessed in our mouse model of VCID-HF. Three-month-old male C57BL/6J mice were subjected to myocardial infarction (MI) to induce heart failure for four weeks and then treated with subcutaneous injections of extended-release PNA5. Microglia activation, BBB permeability, cerebral perfusion, and neurovascular coupling were assessed. Results show that in our VCID-HF model, there was an increase in microglial activation and recruitment within the CA1 and CA3 regions of the hippocampus, a disruption in BBB integrity, and a decrease in neurovascular coupling. Treatment with PNA5 reversed these neuropathological effects of VCID-HF, suggesting that PNA5 may be an effective disease-modifying therapy to treat and prevent VCID. This study identifies potential mechanisms by which heart failure may induce VCID and highlights the possible mechanisms by which treatment with our novel glycosylated Angiotensin-(1-7) Mas receptor agonist, PNA5, may protect cognitive function in our model of VCID.

## INTRODUCTION

Vascular contributions to cognitive impairment and dementia (VCID) and mixed dementia significantly contribute to the 55 million people worldwide who suffer from dementia. Mechanisms thought to contribute to cognitive impairment in patients at risk for VCID include oxidative stress, inflammation [1], decreased cerebral blood flow (CBF) and hypoxia [2, 3], altered cerebrovascular autoregulation [2], and micro embolism [3]. Decreased brain perfusion in cardiac disease, such as that seen in heart failure (HF), has been attributed to low cardiac output, low blood pressure, and altered cerebrovascular reactivity [4, 5], leading to the development of VCID. The ideal therapeutic agent to treat inflammation-related neurodegenerative disease and prevent VCID in persons with cardiovascular disease or hypertension would be designed to interrupt the inflammatory cascade at both sides of the blood-brain barrier, the brain vascular endothelium, and neuronal and glial cells, thus decreasing brain ROS production, reducing neuroinflammation, and improving cerebral vascular function and blood flow.

The neurovascular unit (NVU) is important for maintaining a healthy blood-brain barrier (BBB) and is essential to meeting localized brain oxygen and nutrient demands [6-8]. Previous studies have demonstrated that neurovascular coupling (NVC) activity is blunted in those with dementia and HF [9, 10]. Further, oxidative stress and inflammation also play a critical role in endothelial dysfunction, cerebral microvascular impairments, and blunted NVC responses [11-13]. In addition to oxidative stress, brain inflammation has been shown to impact neurovascular function and increase blood-brain barrier (BBB) permeability [14].

Our research group has developed a neuroprotective treatment approach to VCID induced by HF (VCID-HF). The peptide PNA5 is a novel pleiotropic anti-inflammatory Angiotensin-(1-7) (Ang-1-7) peptide derivative acting at the Mas receptor that has outstanding brain penetration and enhanced bioavailability. PNA5 decreases brain and cerebrovascular inflammation and restores cognitive function in our preclinical VCID model [15-20], likely by reducing endothelial reactive oxygen species (ROS) generation, decreasing brain and circulating inflammatory cytokines, and circulating neurofilament light protein (NfL), a well-known biomarker for neurodegeneration [15, 16, 21, 22]. Within the brain, the Mas receptor is expressed on neurons, glia, and vascular endothelial cells [23]. Activation of Mas decreases ROS and brain inflammation [24-26], increases induction of neuroprotective cytokines, and decreases pro-inflammatory cytokines [24, 27, 28]. Because the Mas receptor is found in high quantities within the hippocampus and perirhinal cortex as well as in vascular endothelial cells, we propose that PNA5 will be particularly effective in targeting memory impairments associated with hypoxia and inflammation-related neurodegenerative disease [21], such as VCID. Given that the BBB integrity, microglia activation, and cerebral blood flow are closely linked to neurodegenerative diseases such as VCID, Alzheimer’s disease, and mixed dementia [29-31], the purpose of the present study was to test the hypothesis that our model of VCID-HF will exhibit increases in microglia activation, decreased BBB integrity and decreased NVC as compared to healthy Controls. Further, we tested the hypothesis that the neuroprotective effects of PNA5 are accompanied by reduced microglia activation, improved BBB integrity and improved cerebrovascular reactivity in our mouse model of VCID-HF.

## MATERIALS AND METHODS

### Experimental Design

#### Justification and minimization of animal usage

All use of animals in this study conformed to the guidelines approved by the Institutional Animal Care and Use Committee at the University of Arizona and in accordance with the National Institutes of Health Guidelines for the Care and Use of Laboratory Animals. Both the number of animals used, and their suffering were minimized. The number of animals needed for each experiment was determined using a G*Power analysis [32]. Importantly for the present study, we have shown in previously published studies that treatment of healthy control mice with daily injections of PNA5 or the native peptide Ang-(1-7) at 1mg/kg/day has no effect on heart function, cognitive function or circulating cytokines [15, 21]. Thus, to minimize the number of animals used in this study and to test the primary hypothesis our animals were randomly assigned to three main groups: 1) Control (sham)-vehicle treated, 2) VCID-HF- vehicle treated, and 3) VCID-HF-PNA5 treated animals. For the present study, three-month-old adult male C57BL/6J mice (starting with n=15 group+15% attrition, purchased from Jackson laboratories) were housed 3 per cage in a temperature and humidity-controlled standard facility. The facility maintained a 12-hour light/dark cycle. Mice had access to water and standard mouse chow *ad libitum*.

#### Synthesis of Peptides

The extended-release formulation of PNA5 (PNA5) was manufactured by Pace Labs, San Diego, California. In brief, PNA5 peptide (PolyPeptide Group) and polylactic-co-glycolic acid (PLGA) (PLGA-752S, Sigma) were both dissolved in dimethyl sulfoxide (DMSO, Sigma) (55% solvent, 40% PLGA) resulting in a 50 mg/ml PNA5 injectable in-situ gel formulation for subcutaneous injection. Control animals received the vehicle DMSO.

#### Experimental Timeline and Peptide Treatment Regimen

**Figure 1.**
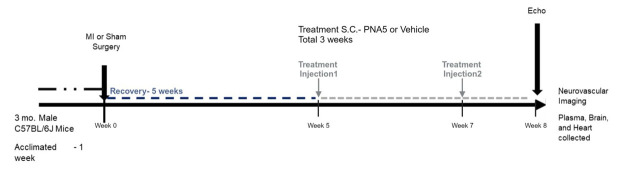
**Experiment timeline**. Three-month old male mice were acclimated for one week before MI or Control surgeries. 5 weeks following recovery, mice were treated with two subcutaneous injections of either Vehicle or PNA5. The first treatment was 5-week post MI surgery, and the second treatment was 7-weeks post MI surgery. One week Following the second treatment, mice were anesthetized for neurovascular imaging, During the last 3 days of treatment mice underwent echocardiograms. They were then anesthetized for neurovascular imaging upon euthanasia plasma, brain, and heart tissue were collected for analysis. MI= myocardial infarct, S.C.=subcutaneous injection.

Figure 1 illustrates the experimental timeline. Following random assignment to one of three treatment groups, animals underwent surgery to induce myocardial infarction to induce HF. Five weeks following recovery, mice were treated with either the vehicle DMSO or PNA5 for 24 days. During the last day of treatment, mice underwent echocardiograms to measure cardiac function and confirm the presence of HF. Animals were then anesthetized for neurovascular imaging. At the end of the neurovascular imaging studies, animals were euthanized, and plasma, brain, and heart tissues were collected for analysis.

### VCID-HF Mouse Mode

To create the VCID-HF model, mice were weighed prior to surgery and anesthetized with 2.5% isoflurane in a mixture of air and O_2_. Left coronary artery ligation was used to induce myocardial infarction (MI) using the procedure described [15, 21]. In short, a left sided thoracotomy was performed at the fourth intercostal space and left anterior descending artery (LAD) was sutured to induce a permanent ligation and MI. Occlusion of the LAD was confirmed by observing myocardial blanching of the left ventricular anterior wall and apex. Control mice underwent sham surgeries, undergoing the same procedure except for the permanent LAD ligation.

### Extended Release PNA5 Pharmacokinetics and Injection Protocol

*Injection Protocol:* Mice were randomly assigned to one of the following three treatment groups: Control (Sham surgery treated with vehicle DMSO), VCID-Vehicle (treated with DMSO), and VCID-PNA5 (treated with PNA5). Five weeks following surgery, animals were treated with a single subcutaneous injection of either 1) Vehicle (DMSO) or 2) PNA5 (25 microliters of 50 mg/ml ER-PNA5 for a total of 1.25 mg) and then second injection at seven weeks following surgery. This dose in this experiment was based on extrapolation from our previous studies using 1mg/kg/day injections for 28 days of PNA5 [15],[16] which for a 30-gram mouse is approximately 33 micrograms/day equal to approximately 1mg total over 30 days. In the present study with the extended-release formulation, we injected 1.25 mg twice separated by two weeks. The decision to give a second injection was based on the consideration that our measured t_1/2_ of PNA5 extended-release formulation in mice is approximately 2.9 days. Thus, to ensure adequate blood levels of PNA5 over the entire treatment period, we gave a second 1.25 mg dose two weeks following the first dose.

#### Heart function and morphology in heart failure

*Echocardiography:* Using a Vevo 2100 High-Resolution Imaging System (Visual Sonics, Toronto, ON, Canada) and a 25-MHz transducer, high-resolution transthoracic ultrasound was performed on anesthetized mice. Data were analyzed using Vevo 2100^®^ analytic software (Visual Sonics, Toronto, ON, Canada). Echo-cardiographic images were taken in B mode. The thickness of the left ventricular wall, interventricular septum, cardiac chamber dimensions, left ventricular posterior wall thickness, and left ventricular internal dimension were measured.

*Histomorphology:* Following euthanasia, hearts were collected, cut longitudinally to expose the interventricular septum and both ventricle chambers, and immersed in 10% formalin overnight at 4 ͦ C followed by dehydration in methanol. Dehydrated heart sections were cleared with xylene for 45 minutes then embedded in paraffin overnight. Heart sections of 5 µm were stained with hematoxylin-eosin to confirm left ventricular wall thinning.

### Novel Object Recognition (NOR) test

Evaluation of mice cognition was achieved with the NOR test as described previously [15, 16, 21]. Briefly, mice were habituated to the testing arena for 2 days, 10 minutes per day. During the learning phase, mice were allowed to explore two identical objects for 6 minutes. At the end of the learning phase, the mice were placed back in their home cages for two more hours. After a 2-hour period, mice were returned to the testing arena for the memory testing phase. One of the two identical objects was replaced with a novel object for the memory testing phase. Mice were allowed to explore both the objects for 2 minutes. Testing boxes and objects were cleaned with 70% ethanol between each phase, to prevent olfactory cues. *Analysis:* A discriminatory ratio (Discrimination ratio) was used to score the mice recognition memory. Discrimination ratio was calculated by: Discrimination ratio= time spent exploring the novel object (t_novel_) minus time spent exploring the familiar object (t_familiar_) divided by the total exploration time [15, 21]:

**Equation 1:** Discrimination ratio= (t_novel_- t_familiar_)/ (total exploration time)

A mouse interacting more with the novel object than the familiar object will have a positive Discrimination ratio score. Mice spending more time with the familiar object than the novel object will have a negative Discrimination ratio.

### Laser Speckle Contrast Imaging of Cerebral Hemodynamics

Assessment of basal cerebral perfusion and neurovascular coupling (NVC) measurements were performed using laser speckle contrast imaging using a high-speed, high-resolution PSI-Z system (Perimed, Jarfalla, Sweden). Mice were anesthetized and intubated for mechanical ventilation, placed in a stereotaxic frame, and an incision was performed in the midline of the scalp to reveal the skull at the whisker somatosensory area [33, 34]. A thinned skull cranial window was performed over the whisker barrel cortex on the contralateral side to the stimulated whiskers. Tests were carried out on mice that were anesthetized with <1.75% isoflurane in order to keep mice sufficiently anesthetized while maintaining near-physiologic cardiovascular function and allowing for stimulated induced hemodynamic responses [35, 36]. All analyses of perfusion were performed using the Perimed software Pimsoft, v2.3.

#### Basal perfusion

Basal perfusion was measured over a one-minute time period where the baseline was consistent. To calculate basal perfusion, we perform three separate analyses 1) analysis to measure the basal perfusion as a measurement of the entire exposed left hemisphere. The measurement of basal perfusion was then normalized to the area that was measured. 2) We measured the same area over the whisker somatosensory area of the mice by transposing the same area pixel size to each animal over the somatosensory area. 3) The third analysis was to identify bregma to measure the basal perfusion within a box with the following parameters, 2 mm caudal from bregma, 2 mm left of bregma, 1.5 mm perpendicular from the midline descending line from bregma.

#### Neurovascular coupling following somatosensory stimulation

Functional cerebral hemodynamics during somatosensory stimulation was assessed after measurement of basal perfusion, as described previously [33]. During acquisition, mice were allowed to stabilize for 3 minutes to record a baseline; following, mice underwent a series of 4 stimulations that lasted 20 seconds. During the first three stimuli the mouse’s contralateral whiskers were repeatedly manually brushed for 20 seconds at a frequency of ~5 Hz. This was repeated 3 times with one-minute intervals between stimulations. This was followed by a negative control as the 4^th^ stimulation, where the ipsilateral whiskers were stimulated for 20 seconds at the end of the experiment. Previous studies demonstrate that manual and mechanical whisker stimulations have similar results [37]. NVC was calculated as a percent change in perfusion from baseline induced by stimulation. Represented values of NVC are the average of the first 3 contralateral whisker stimuli.

**Equation 2:** ((Mean Perfusion During Stimulation)- (Mean Perfusion During Pre-Stimulation)) *100% (Mean Perfusion During Pre-Stimulation)

NVC was calculated by two methods, 1) by quantifying perfusion of individual vessels in the somatosensory area and 2) by measuring the perfusion of a set area over the whisker somatosensory area. This allowed for the investigation of both the most reactive vessels and the average reactivity in the whisker somatosensory area. When looking at the NVC response of individual vessels, the most reactive vessel was measured in each animal. When evaluating NVC by looking at the whisker somatosensory area, the same area was measured over the left whisker somatosensory area for each animal.

### Microglia Analysis

#### Iba-1 staining

Brains were fixed in 4% formalin in sucrose and sectioned at 14 µm-thick sections -1.5 to -2.1 mm from bregma. Free-floating sections were incubated with Iba-1 (Abcam, Catalog#: ab 178847, Lot#:GR3229566-23, 1:500) overnight at 4^o^C. Brains were then stained with secondary (Vector Laboratory, Catalog#: BA-1000-1.5, Lot #: ZJ0930, 1:200) for 2 hours at room temperature and developed via DAB (Vector Laboratories, Catalog#: SK-4100) for 2.5 minutes. Negative controls were incubated with only secondary antibody and developed with DAB. No artificial staining was observed. Brains were imaged and stitched at 40x using the Leica DMA6000 scope and software. For the microglia immunohistochemistry of Iba-1, the control animals were treated with saline (n=3) due to the allocation of the Control-DMSO animals to the BBB portion of the study.

**Figure 2.**
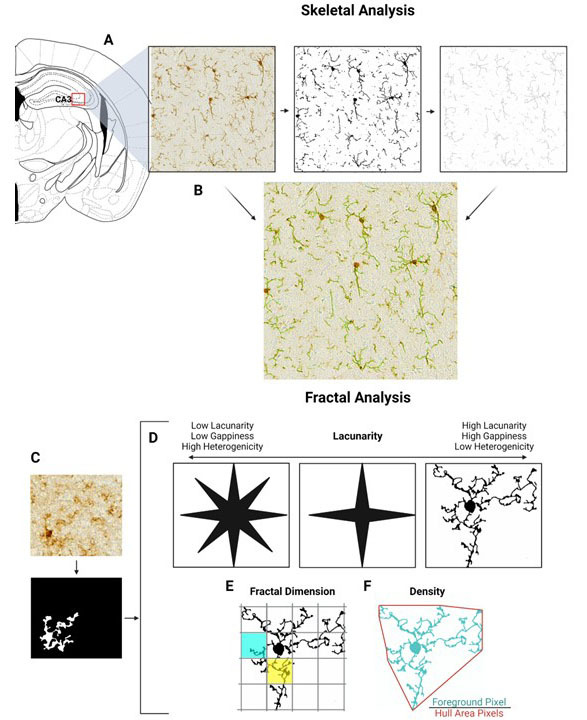
**Visualization of Skeletal analysis and Fractal analysis**. (A and B) represent image processing for skeletal analysis using ImageJ. A) Regions from the CA3 were captured using Leica DMI600 scope and Las X software. Images were then transformed into binary images to be skeletonized using ImageJ. (**B**) Overlay of the skeletonized image (green) and the original image show that skeletonized images represent the processes that are observed in the original image. For these analyses, the following numbers were used: Control n=3 animals (each with 2 to 3 sections per animal), VCID-Vehicle n=4 animals (each with 1-3 sections per animal), VCID-PNA5 n=4 animals (each with 2-3 sections per animal). Fractal analysis of individual cells was performed using FracLac on ImageJ. (**C**) Images of 12 individual cells from the CA3 region per animal were converted into a polarized image. The treatment groups had the following cell numbers: Control n=3 animals, 12 cells per animal. VCID-Vehicle n=4 animals (12 cells per animal, 48 cells total), VCID-PNA5 n=4 animals (12 cells per animal, 48 cells total). FracLac was then used to identify morphologic features of microglia within the three treatment groups. (**D**) Lacunarity refers to gappiness and heterogeneity. With an increase in gappiness or a decrease in homogeneity, there is an increase in lacunarity value. (**E**) Fractal Dimension (DB) counts grid boxes that contain foreground and the pixels within the grid boxes to measure a cell’s complexity. The yellow box has a greater number of foreground pixels compared to the blue box. F) Density = Foreground Pixels/Hull Area. The foreground is represented in teal, and the convex hull area is indicated by a red line.

#### Iba-1 positive cell count

*Hippocampal Microglia*: Iba-1 positive microglia were counted in the CA1 and CA3 region of the hippocampus. The size of region of interest for both the CA3 and CA1 was the same for all images with a set area of 129k pixels isolated from stitched images. All cell count values were log transformed. For each animal, three brain sections were analyzed and brain sections between -1.5 mm to -2.1 mm from bregma were averaged so that each data point represents one animal.

*Meningeal Macrophages:* Iba1 positive microglia/ macrophages were also counted in intact meninges. The number of cells counted was normalized to the length of meninges in the region of interest for each brain section. Only intact miniges that had no folds and were attached to the brain were analyzed. Three regions of meninges were analyzed: one on the dorsal, lateral, and ventral sides. Each region of meninges was averaged per section. All sections for each animal were averaged so that each data point represented one animal. All brain sections were obtained between -1.5 to -2.0 mm from bregma.

#### Microglia Skeletal analysis

Skeletal analysis was performed as previously described [38-41] to measure branch length. Shorter branch length has been suggested as an indication of microglial proinflammatory activation. In brief, using ImageJ (1.53t), images of the CA3 and CA1 regions of the brain were transformed to binary and then skeletonized (Fig. 2A). Figure 2B shows a green overlay of skeletonized image over the original. Images for skeletal analysis were isolated from stitched images of the sectioned brains and contained at least 6 cells per image.1-3 brain sections were measured for each animal. All brain sections were between -1.5 to -2.0 mm from bregma.

#### Microglia Fractal analysis

Fractal analysis was performed as previously published using FracLac [38-40, 42]. This technique assesses microglial processes' complexity, heterogeneity, density, and thickness. Individual cells imaged in the CA3 and CA1 region were transformed into a black-and-white image (Fig. 2C), and morphologies were then analyzed using FracLac on ImageJ (1.53t). Twelve (12) cells were analyzed per animal. FracLac identifies several morphometric parameters (Fig. 2D-F). Lacunarity: refers to gappiness and heterogeneity. Figure 2D shows a representative image of an increase in gappiness and an increase in lacunarity value. Additionally, a loss in homogeneity results in an increase in lacunarity values. It is proposed that cells progressing towards activated inflammatory states may increase complexity and gappiness as the cell enters an intermediate morphological state [43]. Fractal Dimension (DB): measures complexity by counting the grid boxes that contain foreground (the cell) pixels in addition to counting how many foreground pixels are found within each box (Fig. 2E). The yellow box has a greater number of foreground pixels compared to the blue box. Density is calculated as Foreground Pixels/Convex hull area. The foreground is represented in teal, and the convex hull area is indicated by a red line (Fig. 2F).

### Assessment of BBB Integrity

BBB integrity was determined using a protocol previously published by others [44]. In brief, animals were injected intraperitoneally with dextran conjugated to fluorescein 3000Da (FITC 3kDa, Invitrogen, Catalog#: D3306, Lot#:2454233, at 2mM,100μl) and dextran conjugated to tetramethyl rhodamine 10,000Da (TRITC 10kDa, Invitrogen, Catalog#: D1817, Lot#: 2441358, at 2mM, 100μl). Dextran was allowed to circulate for 15 minutes, after which mice were perfused with 15 milliliters of warm PBS over 3 minutes via cardiac perfusion. Brains were separated so that one hemisphere was frozen in OCT for immunohistochemistry, and the other half was frozen for BBB assay. Prior to freezing, each hemisphere of the brain was weighed. Plasma from each animal was also collected and frozen. For the assay, plasma samples were diluted with PBS (30μl PBS + 20μl Plasma) and were stored at -80°C until further processing.

**Figure 3.**
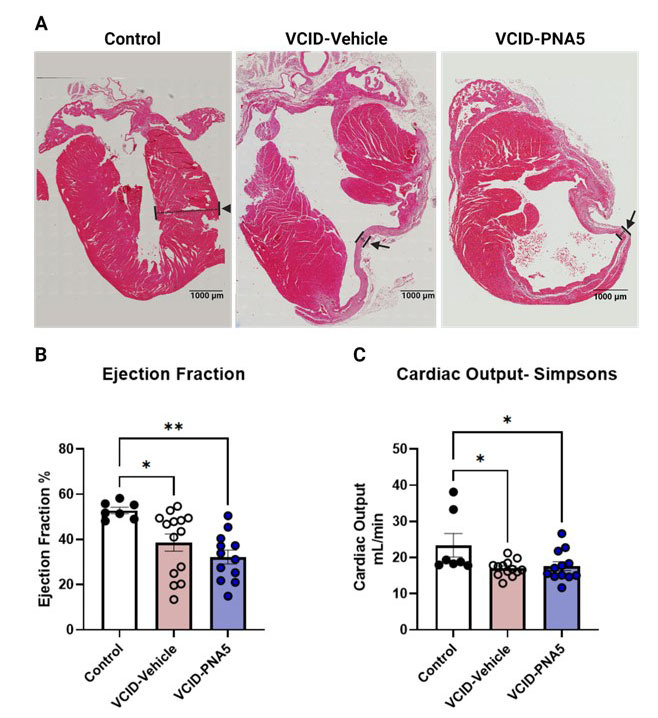
**VCID-HF Mice Exhibit Left Ventricular Wall Thinning and Decreased Ejection Fraction**. (**A**) Hematoxylin and Eosin stains of the heart show thinned left ventricular walls, indicated by the arrow. Cardiac function, assessed as Ejection Fraction (B) and Cardiac Output (C), is reduced in our model. Ejection fraction is calculated through the Simpson method and represented as a percentage of three treatment groups, including Control (n=7), VCID-Vehicle (n=14), and VCID-PNA5 (n=12). Echocardiograms are taken using a Vevo 2100 High-Resolution Imaging System (Visual Sonics, Toronto, ON, Canada) and a 25-MHz transducer. Each point represents an individual animal. Data sets were tested for normal Gaussian distribution via Shapiro-Wilk test. Significance was determined by Kruskal- Wallis test, followed by Dunn’s multiple comparisons for both ejection fraction and cardiac output. Significance was set at a p-value less than 0.05, *p<0.05, **p<0.01. Mice that did not show morphological or functional changes from MI were excluded from VCID groups.

#### Assay

Flash frozen brains protected from light were homogenized in RIPA buffer. Brain lysates and plasma samples’ raw fluorescent index were measured by spectrometer (Biotech) at the excitation/ emission of 490/520 (FITC) and 550/580 (TRITC), respectively (50 µl of sample per well). Raw fluorescence units (RFU) were used to calculate the BBB permeability. Values were normalized to their weight and the treatment group’s plasma sample fluorescence.

**Equation 3** *(Tissue RFU/ Tissue weight in g)/ (Treatment groups averaged plasma RFU)*

#### BBB Immunofluorescence Measurements

Frozen brains were sectioned at 12 µm-thick sections -1.5 to -2.1 mm from bregma and fixed with 1% PFA. Fixed brains were stained with rabbit anti-CD31 (BD bioscience, Catalog#: 553370, Isotype: Rat IgG2a, Lot#: B313252, 0.5mg/ml, clone MEC 13.3, 1:100) for 1.5 hours at room temperature [45]. Brains were stained with secondary anti-rabbit cy5 (Biotium, Catalog#:89138-520, Lot#18C1120, 1:500) labeled for 1 hour at room temperature. Brains were subsequently stained with DAPI (Thermo Scientific, Catalog#: 62248, Lot#: WB3203551, 1:1000) for 30 minutes before mounting with coverslips. Negative controls were incubated with only secondary antibody, followed by DAPI staining. Brains were then imaged and stitched at 40x using the Leica DMA6000 scope (all individual images were captured with the following exposure: DAPI 150 msec, TRITC/FITC 3 seconds, and CD31 1 second). Intensities were measured in ROIs within the CA3, and CA1 regions of the hippocampus.

### Statistical Analysis

Mice that did not show heart morphological or functional changes eight weeks post-MI were excluded from HF groups. GraphPad Prism 9.0 was used to analyze data, and values are represented as mean ± SEM. Data sets were tested for normal Gaussian distribution for all data sets via Shapiro-Wilk test as it is an appropriate test for group sizes smaller than 50. ANOVA followed by the appropriate *post hoc* group comparisons were used to identify statistically significant differences between multiple treatment groups with significance set at p-value <0.05. If the data set had one or more treatment groups that were non-gaussian, data were tested for significance via Kruskal-Wallis test followed by Dunn’s multiple comparisons. Values that were two standard deviations away from the mean were considered outliers and excluded. Significance was set at p- value <0.05. The association between NVC and BBB permeability was analyzed using simple linear regression. The best-fit line for correlations was generated via linear regression and Pearson's correlation. Investigators were blinded for Heart and cognitive evaluations, microglia analysis, BBB assessment and NVC experiments. All n represents the number of biological replicates. Technical replicates were averaged per animal.

## RESULTS

### Extended Release PNA5 Pharmacokinetics (PK)

In collaboration with Pace Labs in San Diego, California we have developed a long-lasting PNA5 in-situ forming gel formulation that results in sustained PNA5 therapeutic blood levels for over 7 days following a single subcutaneous injection (unpublished observations). For the present study, the estimated PK parameters (Excel and PKSolver software) of 1.2 mg of ER-PNA5 injected twice at 2-week intervals were determined in 3 naïve, healthy male mice. Blood samples were obtained at 1 hour post injection and then weekly for 4 weeks. In these studies, the average half-life (t_1/2_) of PNA5-ER in mice was 2.98+0.5 days and the AUC_0-28days_ was 107.4+76 ng/ml*week (n=3). This is compared to the PK values in rats following a single injection of 10 mg/kg PNA5 formulated in saline that we previously reported as having a t_1/2_ of approximately 1.06+0.2 hours [15].

### HF validation through loss of ventricular function

We have previously demonstrated that cardiac function is significantly decreased by 4 weeks post-MI [15, 16, 21]. VCID mice have thinned left ventricular walls, as established in previous literature following MI [46, 47]. As illustrated in Figure 3A, hematoxylin and eosin staining of the heart showed thinned left ventricular walls, indicated by the arrow. Using echocardiography to measure ventricular function, VCID mice treated with either vehicle or PNA5 had significantly lower ejection fraction as compared to Controls (Fig. 3B). Similarly, cardiac output was significantly decreased in VCID mice and was unaffected by PNA5 treatment (Fig. 3C). These results indicate that PNA5 beneficial effects observed within the study were independent of total cardiac function.

### Confirmation of HF-Induced Cognitive Impairment and Rescue effects of PNA5

**Figure 4.**
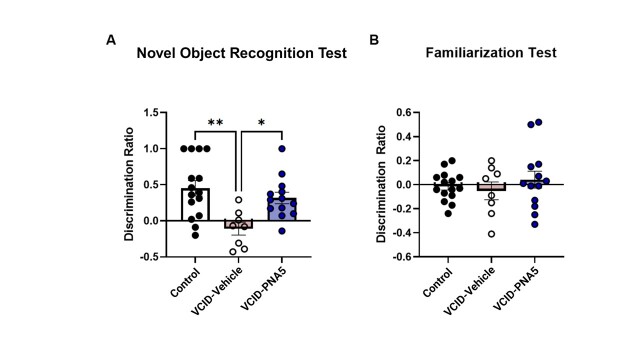
**PNA5 rescue cognition in HF-induced VCID: (A) Memory was tested via novel object recognition test and represented as a discrimination ratio of the following three groups: Control (sham mice that received DMSO as control), VCID-Vehicle**. PNA5 rescues decreases the discrimination ratio observed in VCID mice. D ration is indicative of cognitive ability in memory. Control, 0.451, ± 0.106, n=15 vs. VCID-Saline, -0.109, ± 0.089, n= 8, VCID-PNA5, 0.317, ± 0.079, n=13. (**B**) No differences were observed in the familiarization test. Control, 0.451, ± 0.106, n=15 vs. VCID-Saline, -0.109, ± 0.089, n= 8, VCID-PNA5, 0.317, ± 0.079, n=13. Significance was determined using a one-way ANOVA, followed by Tukey’s multiple comparisons tests for both the novel object recognition test and the familiarization test. Mice that did not show morphological or functional changes from MI were excluded from VCID groups. Significance was set at a p-value less than 0.05, *, **, ***.

Our laboratory has previously published and confirmed using both the Morris Water Maze and Novel Object Recognition (NOR) testing [15, 16, 21] that our model of HF in mice results is cognitive impairment that is reversed by treatment with Angiotensin-(1-7) MasR agonists. In the present study, we confirmed for this study that the animals used in the assessment of microglia activation, BBB integrity and neurovascular coupling showed cognitive impairment using our previously validated NOR test. Mice with intact cognitive abilities have a positive discrimination ratio, while mice with impaired cognitive function may have a negative, or zero Discrimination ratio. In the present study, as we have previously shown, HF results in an impairment of cognitive function as demonstrated by a negative Discrimination Ratio score, which was significantly lower than Control mice. As demonstrated previously, treatment with the Ang-(1-7) MasR agonist PNA5 rescued the cognitive function (Fig. 4A) (Control, 0.451±0.106, n=15 vs. VCID-Saline, -0.109±0.089, n= 8, VCID-PNA5, 0.317±0.079, n=13 Significance was determined via one-way ANOVA, Tukey's multiple comparisons tests). No differences were observed in the familiarization test showing that mobility and exploration were not differently impacted across all three treatment groups (Fig. 4B) (Control, 0.451±0.106, n=15 vs. VCID-Saline, 0.109±0.089, n= 8, VCID-PNA5, 0.317±0.079, n=13. Significance was determined via one-way ANOVA, Tukey's multiple comparisons tests). These data corroborate our previous findings that our model of HF results in decreased cognitive function [15, 16, 21], and that PNA5 is efficacious in preventing cognitive decline in this model.

### PNA5 reverses VCID-associated microglia morphological changes

#### Microglia cell count

**Figure 5.**
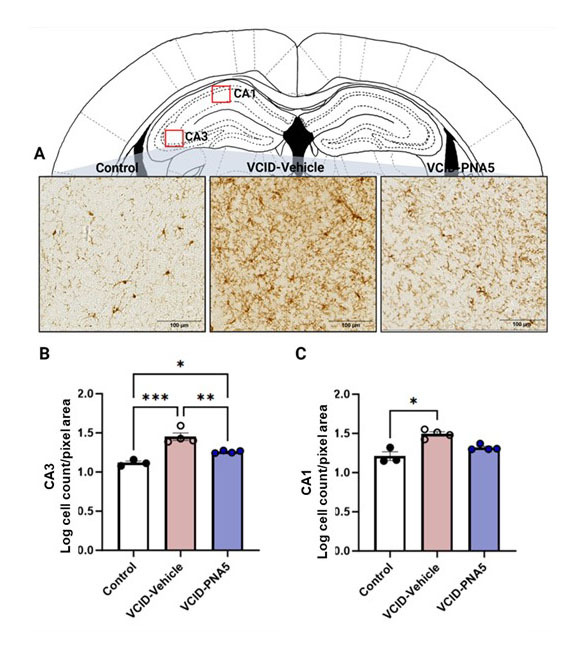
**PNA5 Decreases the Number of Microglia in the Hippocampus**. (**A**) Immunohistochemical analysis of Iba-1 staining. Sections of the brain imaged were located between -1.5 to -2.1mm and were stained with Iba-1 (ab 178847, 1:500). Scale bar equals 100µm. Quantitative analysis of Iba-1 staining of microglia soma count per frame in the (B) CA3 and C) CA1 regions of the hippocampus. Values are represented as log transformation of the number of cells in a region of interest size that was the same for all sections analyzed (region of interest was 12.9k pixel area). Significance was determined using ANOVA followed by Tukey's or a Kruskal-Walli’s test followed by Dunn’s multiple comparisons. CA3: VCID-Vehicle, 1.45 ±0.04 (n=4) vs Control, 1.12 ±0.02 (n=3) p=0.0003; VCID-PNA5, 1.26±0.008 (n=4) vs VCID-Vehicle, p=0.006; Significance was determined via one-way ANOVA. CA1: VCID-Vehicle, 1.46±0.02 (n=4) vs Control 1.21±0.05 (n=3) p=0.031; VCD-PNA5, 1.32±0.01 (n=4) vs VCID-Vehicle, p=0.129; Normality was tested for using Shapiro-Wilk test, and significance was tested using Kruskal- Wallis test, followed by Dunn’s multiple comparisons as these data set were not gaussian. Each point represents an individual animal. Each animal had up to 3 sections analyzed and averaged. Significance was set at p<0.05, *p<0.05, **p<0.01, ***p<0.001.

In this study, we utilized Iba-1 immunohistochemistry to identify and examine microglia within the brain parenchyma in three animal groups including VCID-vehicle (DMSO,n=4), VCID-PNA5 (n=4), and Control animals treated with saline (n=3) Positive Iba-1 cells are also known to be found in meninges (mMΦ) and perivascular spaces (pvMΦ) and Iba-1 is used to identify both microglia and macrophages in the brain [48]. We found that VCID-HF increases microglia cell numbers in hippocampal CA3 and CA1 regions, and PNA5 treatment mitigates these effects. Figure 5A illustrates the Iba-1 staining of microglia in the CA3 region of the hippocampus. Values were represented as log transformation of the cell counts in each area examined. VCID-Vehicle animals had significantly increased numbers of microglia compared to Controls in CA3 and the CA1 regions of the hippocampus. (CA3: VCID-Vehicle, 1.4 ±0.04 (s, n=4) vs Control, 1.1 ±0.02 (n=3) p=0.0003. Significance for the CA3 was determined using a one-way ANOVA followed by post hoc test. CA1: VCID-Vehicle, 1.4 ±0.02 (n=4) vs Control 1.2 ±0.05 (n=3) p=0.03. Significance was determined for CA1 values using a Kruskal- Wallis test followed by a Dunn’s multiple comparison.). (Fig. 5B, 4C). PNA5 treatment significantly decreased the microglia cell number as compared to VCID-Vehicle animals 40% in the CA3 region (CA3: VCID-PNA5, 1.26 ±0.008 (n=4) vs VCID-Vehicle, 1.45 ±0.047 (n=4) p=0.006. Significance for the CA3 was determined using a one-way ANOVA followed by post hoc test.) In the CA1 region, treatment with PNA5 decreased the microglia cell number by 32%, but this did not reach significance. (CA1 VCD-PNA5, 1.32 ±0.018 (n=4) vs VCID-Vehicle, 1.49 ±0.027 (n=4) p=0.129, Significance was determined for CA1 values using a Kruskal- Wallis test followed by a Dunn’s multiple comparison.).

#### Microglia morphological analysis

**Figure 6.**
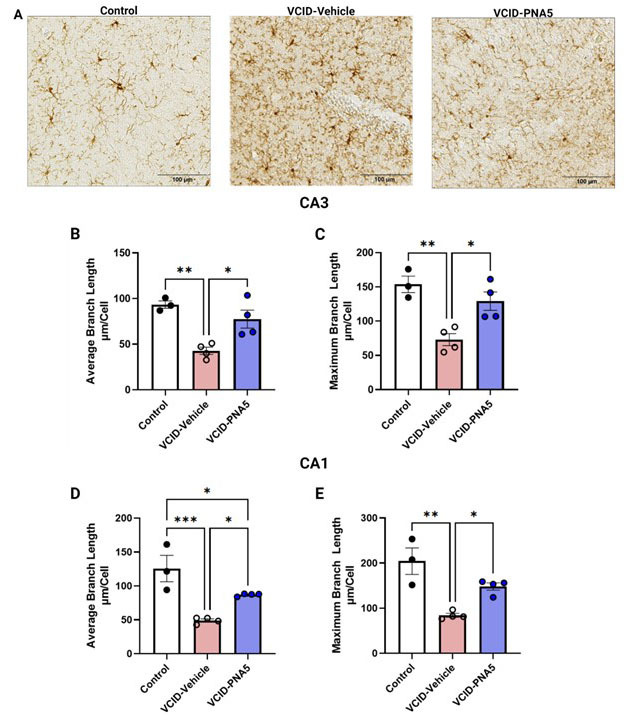
**PNA5 Restores Microglia Branch Length**. (**A**) Immunohistochemical analysis of Iba-1 staining depicts shorter branch length in VCID-Vehicle mice compared to both Control and VCID-PNA5 treated mice. Quantitative analysis of skeletonized images analyzed branch length. CA3 (B), and CA1 (D) average branch length per cell and CA3 (C), and CA1 (E) maximum branch length per cell graphicly show that VCID mice have shorted branch length. Each point represents an individual animal. Control n=3 animals (each with 2 to 3 sections per animal) VCID-Vehicle n=4 animals (each with 1-3 sections per animal) VCID-PNA5 n=4 animals (each with 2-3 sections per animal). CA3- Average branch length: Control 93.4±4.0, VCID-Vehicle 42.8±4.04, VCID-PNA5 77.4±9.9, ANOVA, Tukey's multiple comparisons test Dunn’s multiple tests; CA3-Maximum branch length: Control 153.8±12.0, VCID-Vehicle 73.0±8.7, VCID-PNA5 129.1±13.4 ANOVA, Tukey's multiple comparisons test Dunn’s multiple tests. CA1-Average branch length: Control 125.7±19.4, VCID-Vehicle 49.0±2.3, VCID-PNA5 87.0±1.0, ANOVA, Tukey's multiple comparisons test Dunn’s multiple tests. CA1-Maximum branch length: Control 204.3±29.4, VCID-Vehicle 84.1±4.3, VCID-PNA5 148.1±8.1 significance was determined Kruskal-Wallis Test with a Dunn’s multiple tests. Significance was set at p<0.05, *p<0.05, **p<0.01, ***p<0.001.

Microglia morphology has been closely associated the cells functionality and activity [42]. To measure changes in microglia morphology, skeletal analysis of the microglia in the CA3 and CA1 regions of the hippocampus was performed using ImageJ to measure the length of the microglia processes. Measurements included maximum branch length and average branch length. Figure 6A-E illustrates that VCID-Vehicle mice had significantly shorter average branch length and maximum branch length as compared to Controls. VCID-Vehicle microglia had a bushy morphology similar to some states of activated microglia, as both the average process length and the longest process lengths in each cell were shorter [49, 50]. PNA5 treatment mitigated these VICD changes in microglia morphology (Table 1, Fig. 6A-E).

**Table 1:** Skeletal analysis of Microglia morphology: Compares the morphometric parameters determined with skeletal analysis, including average branch length and maximum branch length.

	CA3			CA1		
	Control (n=3)	VCID-Vehicle (n=4)	VCID-PNA5 (n=4)	Control (n=3)	VCID-Vehicle (n=4)	VCID-PNA5 (n=4)
**Average branch length**	93.43 ±4.025 p=0.003	42.81 ±4.042	77.45 ±9.913 p=0.017	125.70 ±19.460 p=0.001	49.09 ±2.342	87.00 ±1.039 p=0.034
**Maximum branch length**	153.80 ±12.010 p=0.004	73.04 ±8.760	129.10 ±13.460 p=0.018	204.30 ±29.430 p=0.001	84.17±4.348	148.10 ±8.070 p=0.028

P values are a comparison of each treatment group to VCID-Vehicle. For example, p values under Control, are the comparison of Control vs VCID-Vehicle. CA3- Average branch length: ANOVA, Tukey's multiple comparisons test Dunn’s multiple tests; CA3-Maximum branch length: ANOVA, Tukey's multiple comparisons test Dunn’s multiple tests. CA1-Average branch length: ANOVA, Tukey's multiple comparisons test Dunn’s multiple tests. CA1-Maximum branch length Kruskal-Wallis Test with a Dunn’s multiple tests.

**Figure 7.**
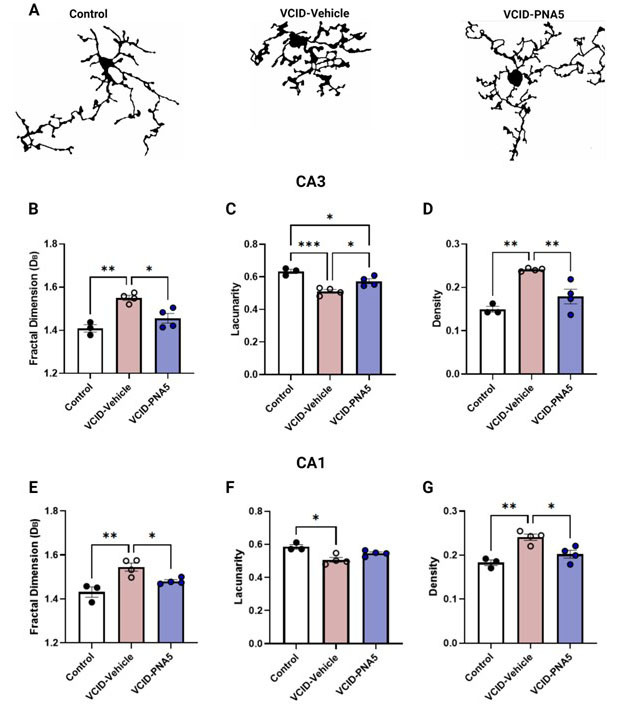
**PNA5 Decreases VCID Associated Microglial Activation**. Morphologic analysis of individual cells of each treatment group were analyzed using FracLac. (**A**) Visual representations of cell morphology are shown for each treatment group, Control (n=3), VCID-Vehicle (n=4), and VCID-PNA5 (n=4). FracLac Identifies several morphometric parameters, including (B, E) Fractal Dimension (DB), a measurement of complexity, C, F) lacunarity, and D, G) density. (For descriptions of each morphological parameter, see Figure). Each point represents 12 cells averaged per individual animal. Twelve cells per animal were analyzed and averaged. Significance was determined by one-way ANOVA, or Kruskal-Wallis Test, with Dunn’s multiple tests for those that do not follow Gaussian distribution. The test per graph are as listed: CA3: Fractal Dimension: one-way ANOVA followed by a Tukey's multiple comparisons test, Lacunarity: one-way ANOVA followed by a Tukey's multiple comparisons test, and Density: one-way ANOVA followed by a Tukey's multiple comparisons test; CA1: Fractal Dimension: one-way ANOVA followed by a Tukey's multiple comparisons test, Lacunarity: Kruskal-Wallis Test, with Dunn’s multiple tests, and Density: one-way ANOVA followed by a Tukey's multiple comparisons test. Significance was set at p<0.05, *p<0.05, **p<0.01, ***p<0.001.

We used fractal analysis to quantify the spatial complexity of the individually isolated microglia to elucidate subtle differences within ramified cells. We measured fractal dimension, lacunarity and density of the microglia in the CA3 and CA1 regions in all three groups. VCID-Vehicle mice had increased fractal dimension compared to Control mice in the CA3 and CA1 regions indicating that VCID-Vehicle mice had greater cell process complexity. (Table 2, Fig. 7B, E). Treatment with PNA5 mitigated these effects in the CA3 and CA1 regions as compared to VCID-Vehicle. We also measured changes in microglia lacunarity (Fig. 7C, F). Lacunarity is a measurement associated with the change in the soma in addition to microglial processes. PNA5 treatment restored lacunarity in VCID mice in the CA3 region as compared to VCID-Vehicle (Table 2, Fig. 7C), and also in CA1, although the CA1 data did not reach significance (Fig. 7F). Lower values as seen in VCID-Vehicle mice compared to Control mice infer greater homogeneity in combination with low amounts of differently sized gaps [51]. Lastly, microglial processes density was higher in VCID-Vehicle mice as compared to Controls in both the CA3 and CA1 regions of the hippocampus (Table 2, Fig. 7D, G); PNA5 treatment prevented the increase in process density. Together these data indicate that VCID-HF results in bushier, denser microglia as seen in microglia transitioning into an “activated state” which has been characterized by shorter, swollen, ramified processes with larger cell bodies [51]. PNA5 prevented these morphological alterations observed in VCID-vehicle mice.

**Table 2:** Fractal analysis of Microglia morphology: Compares the morphometric parameters determined with fractal analysis, including fractal dimension (D_B_), Lacunarity, Density.

	CA3			CA1		
	Control (n=3)	VCID-Vehicle (n=4)	VCID-PNA5 (n=4)	Control (n=3)	VCID-Vehicle (n=4)	VCID-PNA5 (n=4)
**Fractal Dimension (DB)** **a measure of cell complexity**	0.15 ±0.005 p= 0.002	1.55 ±0.003	1.46 ±0.023 p=0.011	1.43 ±0.023 p=0.004	1.55 ±0.019	1.48 ±0.007 p=0.048
**Lacunarity** **gappiness and heterogeneity**	0.63 ±0.013 p=0.001	0.51 ±0.011	0.57 ±0.015 p=0.021	0.58 ±0.013 p=0.017	0.51 ±.0.014	0.55 ±0.008 p=0.603
**Density** **Foreground Pixels/ Hull Area**	0.15 ±0.002 p=0.001	0.24 ±0.002	0.17 ±0.017 p=0.005	0.18 ±0.006 p=0.003	0.24 ± 0.007	0.20 ±0.009 p=0.017

P values are a comparison of each treatment group to VCID-Vehicle. For example, p values under Control, are the comparison of Control vs VCID-Vehicle. **CA3**: Fractal Dimension: one-way ANOVA followed by a Tukey's multiple comparisons test, Lacunarity: one-way ANOVA followed by a Tukey's multiple comparisons test, and Density: one-way ANOVA followed by a Tukey's multiple comparisons test; **CA1**: Fractal Dimension: one-way ANOVA followed by a Tukey's multiple comparisons test, Lacunarity: Kruskal-Wallis Test, with Dunn’s multiple tests, and Density: one-way ANOVA followed by a Tukey's multiple comparisons test.

### VCID increases Iba1 positive cells accumulation in cortical meningeal spaces.

**Figure 8.**
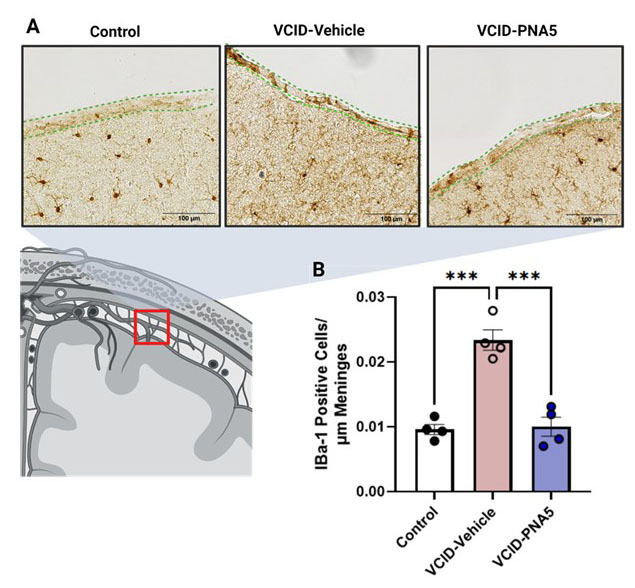
**PNA5 Decreases Iba-1 Immunoreactive Cells in the Meninges**. (**A**) Immunohistochemical analysis of Iba-1 positive cells within meninges in the three groups of mice. Cartoon illustrates (B) Quantification of circulating macrophages in all three treatment groups, Control, VCID-Vehicle, and VCID-PNA5. The number of cells counted was normalized to the length of meninges in the region of interest. Significance was determined by one-way ANOVA. (Control n=4 animals with three brain sections per animal, VCID-Vehicle n=4 animals with three brain sections per animal, and VCID-PNA5 n=4 animals with three brain sections per animal.) (Control 0.01±0.0001, VCID-Vehicle 0.02±0.002, VCID-PNA5 0.01±0.001, one-way ANOVA, followed by Tukey's multiple comparisons test). Each point represents an individual animal. Significance was set at p<0.05, *p<0.05, **p<0.01, ***p<0.001.

The calcium-binding proteins Iba-1 is not only a biomarker for microglia, it is also known to be expressed on macrophages found in the cortical meningeal and subarachnoid regions of the brain [52, 53, 48]. Iba-1 positive meningeal microglia/macrophages have been shown to have increased expression during hypoxic insult, injury, and brain disease [54, 55, 56]. In the present study we examined changes in Iba-1 positive cells in the cortical meningeal regions in our study groups. In addition to changes in microglia number and morphology, VCID-Vehicle treated mice had a significantly higher meningeal Iba-1 positive cell count compared to Controls, (Fig. 8). (VCID-Vehicle 0.021 ±0.002 vs Control 0.01 ±0.001, p=0.0001. Significance was determined via a one-way ANOVA followed by a Turkey’s multiple comparison test.) Treatment with PNA5 resulted in significantly less numbers of Iba-1 positive cells in the meningeal spaces as compared to VCID-Vehicle treated animals (VCID-PNA5 0.01 ±0.001 vs Control 0.01 ±0.001, p=0.0001, one-way ANOVA followed by Tukey's multiple comparisons test). These data suggests that an increase in meningeal inflammation induced by VCID-HF is mitigated by PNA5 treatment.

### PNA5 treatment rescues VCID-induced increase in BBB permeability

**Figure 9.**
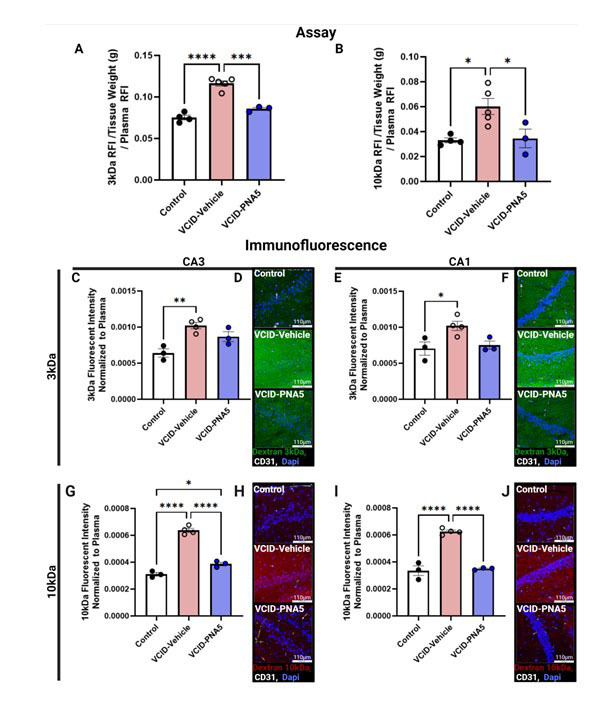
**PNA5 Increases BBB Integrity**. (A, B) Quantification of BBB permeability assay within the three groups. Calculated from assays of brain lysates normalized to plasma dextran levels. 3kDa: VCID-Vehicle (n= 5), 0.12±0.003; 10kDa: VCID-Vehicle (n=5), 0.06±0.006; 3kDa: Control (n=4), 0.08±0.003; 10kDa: Control (n=4), 0.03±0.002. PNA5 prevents VCID-associated increases in BBB permeability of both 3kDa (VCID-PNA5, n=3, 0.09±0.002) and 10kDa (VCID-PNA5, n=3, 0.04±0.007). Significance was determined via one-way ANOVA, Tukey’s multiple comparisons test post. (D, F, H, J) Florescent Histochemistry. Examples Dextran FITC 3kDa fluorescent images at the CA3 (D) and CA1 region of the hippocampus (F), and Dextran TRITC 10kDa at the CA3 (H) and CA1 region of the hippocampus (J) are shown to the left of the corresponding bar graph. The top panel of each representative image is Control, the middle panel is VCID-Vehicle, and the bottom panel are VCID-PNA5 treated animals. Each image was taken at 40x, scale bare (in white) represents 110 µm (all individual images were captured with the following exposure: DAPI 150 msec, TRITC/FITC 3 seconds, and CD31 1 second). Blood vessels identified with CD31 immunohistochemistry (white), and Dapi stained cell bodies (blue). Dextran 3kDa (green), and 10kDa (red). (C, E, G, I) Quantification of Dextran FITC 3kDa and Dextran TRITC 10kDa fluorescence in the CA1 and CA3 hippocampal regions in the three groups. C, D) Dextran 3kDa VCID-HF increased permeability to Dextran 3kDa in both the CA1 and CA3 region of the hippocampus as compared to controls. PNA5 had no significant effect on permeability of Dextran 3kDa. (3kDa CA1: VCID-Vehicle, 0.001 ±6.4e-5 (n=4) vs Control, 0.0007±9.2e-5 (n=3) p=0.04; VCID-PNA5, 0.0008±5.7e-5 (n=3) vs VCID-Vehicle, p=0.07; Significance was tested using one-way ANOVA, followed by Tukey's multiple comparisons test. 3kDa CA3: VCID-Vehicle, 0.001±4.4e-5 (n=4) vs Control, 0.0006±5.8e-5 (n=3) p=0.0; VCID-PNA5, 0.0009±6.8e-5 (n=3) vs VCID-Vehicle, p=0.0; Significance was tested using one-way ANOVA, followed by Tukey's multiple comparisons test) (E, F) Dextran 10 kDa. VCID-HF increased permeability to Dextran 10kDa in both the CA1 and CA3 region of the hippocampus as compared to controls. PNA5 treatment reversed this effect and returned Dextran 10kDa permeability back to levels observed in controls. (10kDa CA1: VCID-Vehicle, 0.0006±1.1e-5 (n=4) vs Control 0.0003±3.6e-5 (n=3) p<0.0001; VCD-PNA5, 0.0003±4.8e-6 (n=3) vs VCID-Vehicle, p<0.0001. Significance was tested using One-way ANOVA, followed by Tukey's multiple comparisons test. 10kDa CA3: VCID-Vehicle, 0.0006±1.5e-5 (n=4) vs Control 0.0003±1.3e-5 (n=3) p<0.0001; VCD-PNA5, 0.0004±1.3e-5 (n=3) vs VCID-Vehicle, p<0.0001; VCD-PNA5vs Control, p=0.02. Significance was tested using One-way ANOVA, followed by Tukey's multiple comparisons test.) Images were analyzed for mean raw fluorescence value. Each point indicates the averaged fluorescence mean values for an individual animal. 2-3 brain sections for each animal were analyzed for each region. Significance was set at p<0.05, *p<0.05, **p<0.01, ***p<0.001.

To determine if the increases in microglia activation and meningeal Iba-1 positive cells were accompanied by changes in BBB integrity, we measured fluorescence intensity of both 3kDa and 10kDa dextran in whole-brain hemisphere lysates and brain slices in all three experimental groups. Figure 9A, B illustrate that VCID-Vehicle mice have increased BBB permeability to both 3kDa and 10 kDa fluorescent Dextrans as compared to Controls (3kDa: VCID-Vehicle, 0.11 ±0.003 (n=5) vs Control, 0.08 ±0.002, (n=4), p<0.0001; significance was determined via a one-way ANOVA followed by a Turkey’s multiple comparison test. 10kDa: VCID-Vehicle, 0.06 ±0.006 (n=5) vs Control, 0.03 ±0.001 (n=4), p=0.016. Significance was determined via a one-way ANOVA followed by a Turkey’s multiple comparison test.) PNA5 treatments restored BBB integrity and decreased BBB permeability as compared to VCID-vehicle treated mice (3kDa: VCID-PNA5 0.09 ±0.002 vs VCID-Vehicle 0.11 ±0.003 (n=5), p=0.0001; significance was determined via a one-way ANOVA followed by a Turkey’s multiple comparison test. 10kDa: VCID-PNA5 0.03 ±0.007, n=3 vs VCID-Vehicle 0.06 ±0.006 (n=5), p=0.034; significance was determined via a one-way ANOVA followed by a Turkey’s multiple comparison test.)

**Figure 10.**
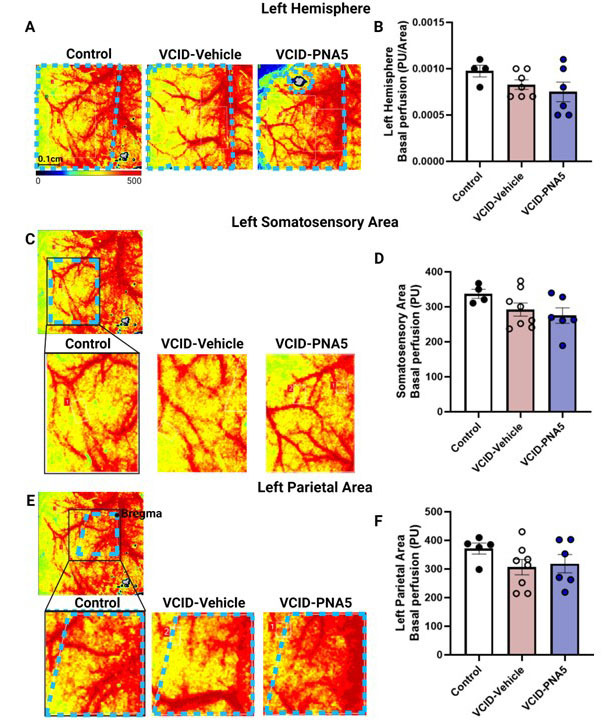
**VCID-HF Has No Effect on Basal perfusion**. (**A**) Heat map represents left hemisphere basal perfusion for Control, VCIDF-Vehicle, and VCID-PNA5. Basal values are taken over the period of ~1 minute. Left hemisphere values are normalized to the measured area. For the somatosensory area (Control n=4, VCIDF-Vehicle n=7, and VCID-PNA5 n=6) C) and the parietal area (Control n=4, VCIDF-Vehicle n=8, and VCID-PNA5 n=6) (E), the size remains the same for each region of interest; hence the values were not normalized to the area. Blue dotted lines outline the measured area. Quantification of the left hemisphere basal perfusion (B), the somatosensory area (D), and the left parietal area (Control n=5, VCIDF-Vehicle n=8, and VCID-PNA5 n=6) (F) show no significant difference between treatment groups. Each point represents an individual animal. Data are represented in perfusion units (PU). Significance was determined by one-way ANOVA. Mice that did not show morphological or functional changes from MI were excluded from VICD groups. Significance was set at p<0.05.

We also assessed regional differences in VCID-HF induced BBB permeability. Figure 9D, and F show representative images of 3kDa FITC-dextran fluorescent images in the CA3 and CA1 regions of the hippocampus, Figure 9H, and J summarizes images that represent 10kDa TRITC-dextran CA3 and CA1 regions of the hippocampus. Regional semi-quantitative immune-fluorescence assessment showed that VCID-HF disrupts the BBB in the CA3 and CA1 regions of the hippocampus, resulting in increased permeability to both 3kDa (Fig. 9C, D) and 10kDa (Fig. 9E, F) dextran, respectively, when compared to Controls. PNA5 treatment significantly decreased permeability to 10kDa dextran in both regions but did not affect permeability to 3kDa dextran.

### PNA5 rescues blunted cerebral functional hyperemia in VCID mice

To determine if VCID-HF induced microglia activation and BBB disruption are accompanied by changes in cerebral blood flow, and whether possible impairments in cerebral hemodynamics are prevented by PNA5, we measured basal brain blood perfusion as well as neurovascular coupling. Basal blood perfusion was evaluated using three methods 1) evaluation of the left hemisphere normalized to area, 2) evaluation of the left hemisphere in the somatosensory area, and 3) evaluation of midsagittal blood perfusion by measuring perfusion in a set area and location from bregma.

#### Basal Brain Blood Perfusion

Heat maps of the left hemisphere, left whisker somatosensory area, and left parietal region indicate that there were no differences in basal blood perfusion in all three treatment groups (Fig. 10) (Fig. 10A, B, Left hemisphere: VCID-Vehicle 8.3x10^-4^ ±5.2x10^-5^ (n=7) vs Control 9.8x10^-4^ ±6.3x10^-5^ (n=4), p= 0.450 and VCID-PNA5 7.5x10^-4^ ±1.1x10^-4^ (n=6) vs VCID-Vehicle 8.3x10^-4^ ±5.2x10^-5^, p= 0.73; Fig. 10C, D, Left somatosensory area: VCID-Vehicle 292.40 ±18.81 (n=8) vs Control 337.20 ±13.03 (n=4), p= 0.32; VCID-PNA5 275.10 ±22.20 (n=6) vs VCID-Vehicle 292.40 ±18.81 p= 0.79; Fig. 10E, F, Left parietal area: VCID-Vehicle 306.70 ±26.74 (n=8) vs Control 371.40 ±19.04 (n=5) p=0.26; VCID-PNA5 318.50 ±32.01 (n=6) vs VCID-Vehicle 306.70 ±26.74 p=0.94, one-way ANOVA followed by Tukey's multiple comparisons test).

#### Neurovascular Coupling

**Figure 11.**
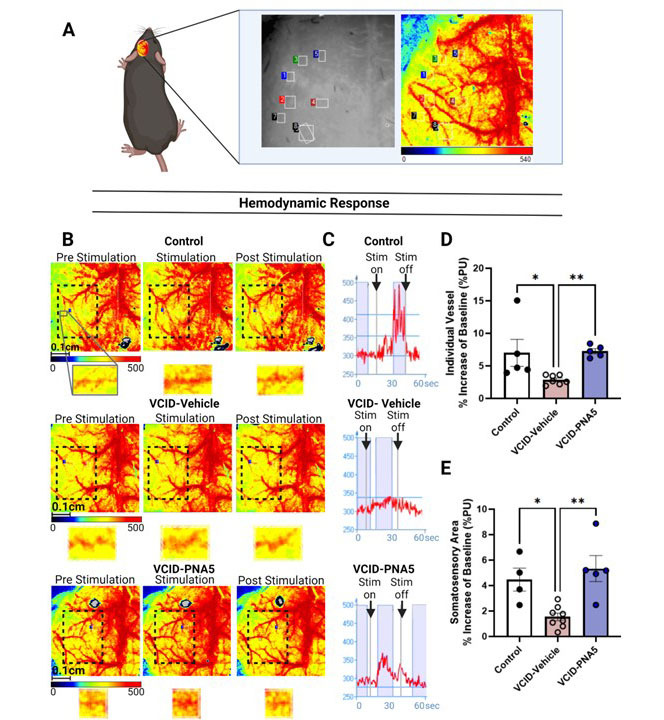
**PNA5 Rescues Neurovascular Coupling (NVC)**. (**A**) is a cartoon description of whisker stimulation, the corresponding heat map, and raw data output that occurs during testing neurovascular coupling. Each colored box (blue, green, red, maroon, and black) on the grey scale image represents regions of interest (ROI) that focus on an individual vessel. The same boxes can be observed in the heatmap to the right of the black and white image. (**B**) Images of the left hemisphere in brain perfusion during pre-stimulation, stimulation, and post-stimulation (images moving from the left to the right) are depicted in the heat map form of the groups. Black dashed lines surround the region of the somatosensory whisker. Underneath each image of the left hemisphere are magnified images of isolated vessels that depict the relative reaction of brain perfusion with stimulation. (**C**) Raw data traces show a peak in the blood perfusion during stimulus over time in seconds of an ROI for an individual vessel. The “Stim on” arrow indicates the start of the stimulus. The “Stim off” arrow indicates the end of the stimulus. Blue bars are examples of times of interest that are sampled for perfusion values. Quantification of changes in NVC response (see Equation 2) for individual vessels in the treatment groups Control (n=4), VCID-Vehicle mice (n=7), and the VCID-PNA5 (n=5) (D) and whisker somatosensory area in the treatment groups Control (n=4), VCID-Vehicle mice (n=8), and the VCID-PNA5 (n=5) (E). Individual vessel: Control 7.00±2.076, VCID-Vehicle mean= 2.87±0.266, HF-PNA5 7.25±0.401 significance was determined using Kruskal-Wallis test followed by Dunn's multiple comparisons test; Somatosensory: Control 4.46±0.901, VCID-Vehicle 1.39±0.298, VCID-PNA5 5.30±1.021, ANOVA, followed by Tukey’s multiple comparisons test for values. Each point represents an individual animal. Data are represented in perfusion units (PU). Significance was set at p<0.05, *p<0.05, **p<0.01, ***p<0.001.

Figure 11A is a cartoon depiction of whisker stimulation, the corresponding heat map, and raw data output that occurs during testing neurovascular coupling. During whisker stimulation, heat map images represent increases in blood perfusion in vessels of the whisker barrel cortex. Increases in blood perfusion are indicated by a warmer color moving from yellow to red (Fig. 11B, the black segmented line surrounds the whisker somatosensory are, zoomed in images show the individual vessel response during whisker stimuli). Whisker stimulation induced the expected increase in blood perfusion atop the whisker barrel cortex in Control mice (Fig. 11B, C, top panels). This response was significantly blunted in VCID-Vehicle mice (Fig. 11B, C, middle panels) and treatment with PNA5 restored the functional hyperemia responses (Fig. 11B, C, bottom panels). These effects are quantified for individual vessels (Fig. 11D) (VCID-Vehicle 2.87 ±0.27 (n=7) vs Control 7.00 ±2.07 (n=5), p=0.037; VCID-PNA5 7.26 ±0.40 (n=5) vs VCID-Vehicle, p=0.0035, n=7, significance was determined using Kruskal-Wallis test followed by Dunn's multiple comparisons test) and, as illustrated in Figure 11E, for the entire somatosensory cortex (VCID-Vehicle 1.32 ±0.26 (n=8) vs Control 4.47 ±0.90, p=0.025, n=4; VCID-PNA5 5.31 ±1.02 (n=5) vs VCID-Vehicle, p=0.003, n=7 significancy was determined using one-way ANOVA, and Dunnett’s post-hoc). Indicating that PNA5 rescues NVC responses in both the most reactive vessels and the average reactivity in the whisker somatosensory area. All test ANOVA, and Dunnett’s post-hoc). Together, these data show that VCID-HF blunts neurovascular coupling responses in the somatosensory cortex and that these responses are rescued by treatment with PNA5.

## DISCUSSION

Our study demonstrates that VCID induced by heart failure results in increased hippocampal microglia activation, decreased BBB integrity, and reduced NVC. Furthermore, treatments with PNA5 mitigated these HF-induced effects by decreasing microglia activation, improving BBB integrity, and restoring NVC. These results identify potential mechanisms by which HF may induce VCID and highlight the possible mechanisms by which treatment with PNA5 may protect cognitive function in our model of VCID.

Currently, there are no approved therapeutics for treating cognitive impairment in patients at risk for VCID. Although precise triggers are debated, diseases that increase systemic and brain inflammation and decrease cerebrovascular perfusions, such as CVD and HF, share common cognitive injuring mechanisms. These mechanisms include increased ROS production, activation of inflammatory pathways, and decreases in blood flow leading to neuronal dysfunction, cognitive impairment, and increased risk for developing VCID [1, 4, 57, 58]. Inflammatory processes play an important role in VCID. Elevated levels of inflammatory markers are associated with cognitive impairment in HF [1]. Several studies have reported reduced performance on episodic and working memory tasks in inflammation [59, 60]. In previous studies, CBF measured with single-photon emission computed tomography was reduced by 30% in patients with severe HF [57]. Decreased perfusion in HF has been attributed to low cardiac output and altered cerebrovascular reactivity [4, 5]. Morphometric MRI studies show decreased gray and white matter volumes in HF patients, particularly in areas critical for memory and executive functions [3, 61], further highlighting perfusion-dependent pathological alterations present in VCID-HF.

### Microglia Activation and HF-Induced VCID

Our results show that VCID-Vehicle mice had increased microglial cell count compared to Control in the CA3 and CA1 hippocampal regions. PNA5 treatments decreased these microglia counts in VCID. During injury or trauma, microglia can migrate to the damaged site [62]. Increased microglial proliferation during neurodegenerative diseases has also been observed in humans with AD and mice with prion disease [63, 64]. Hence, our results of increased hippocampal microglial cell density in VCID-HF may indicate an upregulation of cell migration and proliferation in response to neuronal damage or stress.

In addition to microglial cell count, changes in microglial morphology can be indicative of proinflammatory cell activation. We observed that microglia in VCID-HF vehicle treated mice show morphology similar to intermediate-activated microglia in the hippocampal CA3 and CA1 regions, which were reversed by PNA5 treatment. In the absence of disease or stress, M2 phenotype microglia are predominant; they produce neuroprotective cytokines and can improve neurological function after ischemia [65, 66]. These microglia are important for immune surveillance and maintaining healthy synapses by removing potentially cytotoxic debris. M2 microglia generally have thin cell bodies and processes [67], similar to what we had observed in our Control mice and VCID-PNA5-treated mice. During chronic inflammatory conditions, such as that observed in HF, microglia can transition into M1-activated states [65, 68-70]. Activated microglia have been observed in multiple neurodegenerative diseases, including AD, Parkinson’s, and multiple sclerosis [71]. Situations with hypoxia, systemic inflammation, and trauma, which are observed in HF, have been shown to activate microglia [72, 73]. Activated microglia increases ROS and cytokine production, and initiates brain inflammatory pathways [74-76], contributing to neuronal dysfunction and cognitive impairment.

Our results show that VCID was associated with microglia morphological changes similar to microglia transitioning to the “activated state” [51]. Our VCID microglia assumed a “bushy-like” morphology, with increased density of shorter, thicker, hyper-ramified branches. Thicker, shorter branches have been associated with increased inflammatory activation [42, 51]. Additionally, VCID-Vehicle microglia had significantly higher fractal dimensions than those of Control mice, indicating an increase in cell complexity similar to that observed in activated, immunoreactive microglia [77]. In agreement with our findings, a previous study reported analogous microglial morphological changes in the hippocampus of patients with heart disease [68]. PNA5 prevented microglial activation observed in VCID-HF. PNA5-treated mice maintain elongated, thinner processes like those observed in other studies of resting microglia [51] and our Control mice. Thus, PNA5’s neuroprotective effects may be attributed to mitigating microglial neurodegenerative pathways in VCID-HF.

### PNA5 Decreases VCID-Associated Increase in Meningeal Iba-1 Positive Cells

Additionally, we found that VCID-HF increased Iba-1-positive cells in the brain meninges. It is well known that the calcium-binding protein Iba-1 is not only a biomarker for microglia but is also expressed on macrophages within the subarachnoid spaces and cortical meninges within the brain [54-56]. Recent studies have suggested impaired meningeal lymphatic drainage may contribute to Alzheimer’s disease as an accumulation of Aβ in the brain and may deleteriously affect the BBB and brain blood flow [55, 78, 79].

The meninges can impact the central nervous system (CNS) immune response due to the diverse population of immune cells it hosts [80]. The lymphatics in the meninges are a unique area that allow drained CNS-derived antigens to interface with antigen-presenting cells such as macrophages. These cells can influence the cytokine milieu and parenchymal inflammatory responses [79, 81]. Meningeal lymphatics heavily impact the inflammatory processes and CNS immune surveillance [79]. Iba-1 positive border associate macrophages found in perivascular spaces and meninges have been hypothesized to regulate BBB permeability and maintain lymphatic drainage [82]. We showed that VCID-HF has increased brain meningeal Iba-1-positive cells, and PNA5 treatments reduced Iba-1 cell density in the meninges, suggesting PNA5 may be protective against VCID-HF neuropathology.

### Blood-Brain-Barrier

Our mouse model of VCID-HF has an increased BBB permeability that was restored by treatment with PNA5. The BBB regulates the movement of molecules and cells from the blood to the brain via an endothelial-cell barrier [7, 83, 84]. Previous studies have demonstrated that increased BBB permeability is observed in many types of dementia, including VCID and AD [85, 86]. We propose that the increases in pro-inflammatory milieu [16, 21] and microglia activation in HF-induced VCID disrupts tight junctions within the BBB, leading to neurovascular damage. Studies have shown that activated microglia increase BBB permeability [65]. PNA5 improved BBB function, as observed in the decreased dextran accumulation in the cerebral cortex, suggesting that PNA5 may protect BBB function in VCID-HF.

### Neurovascular Unit and Neurovascular Coupling (NVC)

Our mouse model of VCID-HF has impaired NVC, and PNA5 treatments restored NVC function. The brain has limited intrinsic energy storage and depends on locally regulated hemodynamics for nutrient delivery and removal of metabolites [6]. This hemodynamic responsivity to metabolic needs is facilitated through the neurovascular unit by mechanisms of NVC. Impaired cerebral hemodynamics can lead to chronic hypoxia and, over time, neuronal damage [33]. The hippocampus is susceptible to hypoxia and is often damaged in early AD [87, 88]. This may account for the significant memory impairment observed in our previous studies in our VCID-HF mice [15, 16, 21]. Pro-inflammatory changes observed in VCID, and increased BBB permeability can negatively impact the NVU [9, 15, 89, 90]. Impaired NVC has been previously linked to increased BBB permeability due to endothelial damage [90]. Similarly, we showed that increased BBB permeability and NVC responses had a significant inverse correlation. We propose that increased pro-inflammatory expression may occur at the level of the neurovascular unit in VCID-HF leading to increased BBB permeability, neurovascular damage, and loss of NVC.

We showed that blunted NVC was independent of changed basal perfusion in our VCID-HF model. Chronic hypoperfusion and blunted NVC are observed in patients with chronic HF [57], suggesting that lower NVC responses may be a consequence of reduced global perfusion to the brain. However, patients with mild HF have blunted NVC responses and cognitive decline, despite no basal cerebral blood flow alterations [91]. This suggests that NVC responses can be independent from basal perfusion and are vital for maintaining cognitive health. Similarly, we reported that VCID-HF did not affect basal cerebral perfusion but impaired NVC. PNA5 prevented NVC disruption in VCID mice without changing basal perfusion, further highlighting mechanisms that may underlie PNA5’s neuroprotective effects. The current study, together with our previous publications [15, 16, 21], suggest that PNA5 may rescue NVC by decreasing inflammation and protecting the BBB, thereby preventing further cognitive decline.

Current limitations of our study include that laser speckle imaging has limited penetration within the cerebral tissue; cerebral microvascular hemodynamics is recorded at a depth of ~0.7mm via a thinned-skull cranial window. Hence, we are missing potential changes in basal blood flow and NVC within the hippocampus. Further experiments using functional MRI are needed to address this limitation. Secondly, these studies were only performed in male animals. We have plans to continue these studies in female animals and explore any sex differences in the responses. Finally, to explore longer effects of PNA5 treatment, 6- and 9-month toxicology studies with PNA5 in 2 species are in progress as required prior to beginning PNA5 clinical trials in VCID HF patients.

In conclusion, VCID-HF results in an increase in microglia activation, a decrease in BBB integrity, and a decrease in neurovascular coupling. Further, PNA5 treatment reverses these effects and may be a first-in-class disease-modifying therapy to treat VCID.
